# *Peucedanum ostruthium* Inhibits E-Selectin and VCAM-1 Expression in Endothelial Cells through Interference with NF-κB Signaling

**DOI:** 10.3390/biom10091215

**Published:** 2020-08-21

**Authors:** Christoph Lammel, Julia Zwirchmayr, Jaqueline Seigner, Judith M. Rollinger, Rainer de Martin

**Affiliations:** 1 Department of Vascular Biology and Thrombosis Research, Medical University of Vienna, Schwarzspanierstaße 17, 1090 Vienna, Austria; christoph.lammel@gmx.at (C.L.); jacqueline.seigner@meduniwien.ac.at (J.S.); rainer.demartin@meduniwien.ac.at (R.d.M.); 2 Department of Pharmacognosy, Faculty of Life Sciences, University of Vienna, Althanstraße 14, 1090 Vienna, Austria; julia.zwirchmayr@univie.ac.at

**Keywords:** *Peucedanum ostruthium*, masterwort, inflammation, endothelial cells, E-selectin, VCAM-1, NF-κB

## Abstract

Twenty natural remedies traditionally used against different inflammatory diseases were probed for their potential to suppress the expression of the inflammatory markers E-selectin and VCAM-1 in a model system of IL-1 stimulated human umbilical vein endothelial cells (HUVEC). One third of the tested extracts showed in vitro inhibitory effects comparable to the positive control oxozeaenol, an inhibitor of TAK1. Among them, the extract derived from the roots and rhizomes of *Peucedanum ostruthium* (i.e., Radix Imperatoriae), also known as masterwort, showed a pronounced and dose-dependent inhibitory effect. Reporter gene analysis demonstrated that inhibition takes place on the transcriptional level and involves the transcription factor NF-κB. A more detailed analysis revealed that the *P. ostruthium* extract (PO) affected the phosphorylation, degradation, and resynthesis of IκBα, the activation of IKKs, and the nuclear translocation of the NF-κB subunit RelA. Strikingly, early effects on this pathway were less affected as compared to later ones, suggesting that PO may act on mechanism(s) that are downstream of nuclear translocation. As the majority of cognate NF-κB inhibitors affect upstream events such as IKK2, these findings could indicate the existence of targetable signaling events at later stages of NF-κB activation.

## 1. Introduction

The inflammatory reaction is a common feature of several diseases, including cardiovascular, gastrointestinal, and neurodegenerative, as well as joint and skin disorders. Inflammatory mediators such as IL-1 and TNF or bacterial LPS evoke the expression of a complex set of genes in the endothelium that encode, e.g., chemoattractants and adhesion molecules, a pre-requisite for the adherence and transmigration of immune cells through the vascular wall into the underlying tissue, where they migrate towards the site of injury. However, they also fulfill other functions that are related to proliferation, cell and tissue dynamics, metabolism, survival and apoptosis [1]. Examples of induced genes include the adhesion molecules E-selectin, ICAM-1, VCAM-1, the cytokines IL-1, IL-6, IL-8, chemokines and their receptors, cyclooxygenase, and anti-apoptotic proteins of the IAP family and A20.

The transcription factor NF-κB has been demonstrated to play a major role in the expression of many of these genes, and inhibition of its activation was shown to prevent or at least attenuate pro-inflammatory gene expression. NF-κB comprises a family of five members that can form homo- and heterodimers, the most common one being the RelA/p50 heterodimer. It is held in the cytoplasm through interaction with one of its inhibitors, IκBα, which prevents its nuclear translocation (for review see [2,3]). Following stimulation of endothelial cells with IL-1, TNF or LPS, signals are generated that proceed in part via receptor-specific adapter molecules towards a common kinase complex consisting of IKK1, IKK2 and the regulatory subunit NEMO/IKKγ. Thereby, IKK2 is mainly responsible for the phosphorylation of IκBα, a signal that, in turn, leads to its K48-linked ubiquitination and proteasomal degradation. Subsequently, NF-κB is free to translocate to the nucleus, bind to its respective binding sites in the promoter region of the genes mentioned above, and direct their transcription. Several feedback mechanisms, including NF-κB-dependent IκBα resynthesis, A20 expression, or IKK2 hyper-phosphorylation, ensure a timely shut-down of NF-κB activity and thereby the transient nature of its activation [4,5]. Last not least, regulatory steps affecting the transactivation domain, miRNAs, as well as cross-talk with other pathways, can modulate NF-κB activity [6,7,8].

Despite the availability of numerous steroidal and non-steroidal anti-inflammatory drugs limitations to their use sometimes occur in clinical settings, e.g., in the treatment of chronic diseases, calling for a quest for novel lead structures. Nature has been a rich source of such structures for all kind of maladies, including inflammation [9], and treatment is often backed by long-standing traditional use. A previous investigation on 71 Austrian herbal drugs identified in vitro anti-inflammatory extracts in the so-called “VOLKSMED” database [10] and revealed inter alia the root extract of *Peucedanum ostruthium* (PO) as a promising herbal remedy for a closer investigation. Here, we report the screening of 20 extracts from traditionally used herbal and fungal materials as well as the in-depth characterization of PO, and provide mechanistic insight into its mode of action.

## 2. Materials and Methods

### 2.1. Preparation of Herbal/Fungal Extracts

Plant and mushroom materials were obtained from different suppliers. Detailed information on the used plant/fungal species, organ and the respective extraction solvents are given in Table 1. Voucher specimens are deposited at the Department of Pharmacognosy, University of Vienna, Austria, if not otherwise indicated. Small-scale extracts were prepared according to the protocol for the generation of lead-like enhanced (LLE) extracts as previously described in [11] adapted from [12], if not otherwise indicated (Table 1). Briefly, 0.3 g of dried, pulverized herbal or fungal material was defatted with 5 mL *n*-hexane (VWR International, Radnor, PA, USA; AnalaR NORMAPUR ACS, ≥95%). The remaining material was extracted successively with 7 mL dichloromethane (CH_2_Cl_2_; VWR International; GPR RECTAPUR, ≥99%) and 13 mL methanol (MeOH, AnalaR NORMAPUR ACS, ≥99.8%) at room temperature under sonication for 15 min. The extracts were filtered, combined and dried under vacuum to obtain lead-like enhanced extracts without tannin depletion (LLE w/o TD). Tannin-rich extracts were further subjected to tannin depletion to avoid any unspecific assay interferences. Protocols for the generation of tannin-depleted samples have been described previously [12,13] and successfully applied in several studies [11,14,15,16]. For this process, the dried CH_2_Cl_2_-MeOH extracts were restored in 4-mL MeOH, loaded onto a 3-mL solid-phase extraction cartridge (Phenomenex, Torrance, CA, USA; AH0-7001) filled with polyamide gel (Carl Roth, Karlsruhe, Germany; CC-6; 900 mg) and washed two times with MeOH. The obtained tannin-depleted samples were dried under vacuum to deliver the final extract (LLE). Dried extracts were dissolved in DMSO (Carl Roth; Rotipuran ≥ 99.8%, p.a.) to a final concentration of 10 mg/mL. Samples were stored at −20 °C until used.

### 2.2. Cell Culture

Human endothelial cells (HUVEC) were isolated from the veins of umbilical cords as described previously [19], and maintained in M199 medium (Lonza, Basel, Switzerland, #12-119F) supplemented with 20% FCS (Sigma, St. Louis, MO, USA, #F6765), 2 mM L-glutamine (Sigma; #G7513), penicillin (100 units/mL), streptomycin (100 μg/mL), (Pen-Strep, Lonza, Basel, Switzerland, #DE17-602E), 5 units/mL heparin, and 25 μg/mL ECGS (Promocell, Heidelberg, Germany, ECGS/Heparin #C-30140). For experiments, post-confluent cells of passage no greater than 5 were used.

### 2.3. Antibodies and Reagents

Antibodies were obtained from the following suppliers and used at the respective dilutions: IκBα (Cell Signaling, Frankfurt, Germany, #9241; 1:1000), phospho-IκBα (Cell Signaling, #2859), IKK2 (Cell Signaling, #8943), phospho-IKK1/2 (Cell Signaling, #2694), ß-actin (Santa Cruz, Heidelberg, Germany, #sc-1616), and NF-κB p65 (Santa Cruz #sc-372). As secondary antibodies, goat anti-mouse IgG (HL) cross-adsorbed secondary antibody, HRP (Invitrogen, Carlsbad, CA, USA, #31432), donkey anti-rabbit IgG HRP-linked whole antibody (Sigma, #GENA934), and goat anti-rabbit IgG (HL; highly cross-adsorbed secondary antibody) conjugates with Alexa Fluor 488 (Invitrogen, #A32723) were used. The TAK1 inhibitor (5Z)-7-oxozeaenol: (8-(5-chloro-2-(4-methylpiperazin-1-yl) isonicotinamido)-1-(4-fluorophenyl)-4,5-dihydro-1H-benzo [g] indazole-3-carboxamide was obtained from Sigma (#499610). Recombinant human IL-1ß was from R&D Systems, Minneapolis, MN, USA (#201-LB).

### 2.4. Cytotoxicity Assays

HUVECs were incubated for 4 h with different concentrations of PO (50, 25, 12.5 μg/mL), and the CellTox Green Cytotoxicity Assay (Promega, Madison, WI, USA, #G8741) was performed according to the manufacturer’s recommendations. Cytotoxicity was judged by morphological examination.

### 2.5. Real-Time PCR

Total RNA was isolated using the PeqGold Total RNA Isolation Kit (VWR International, Radnor, USA, #732-2868) according to the manufacturer’s instructions. A total of 1 μg RNA was reverse-transcribed using random hexamers (Fisher Scientific, Schwerte, Germany; #SO142) and murine leukemia virus reverse transcriptase (Fisher Scientific, #10338842). Primers were designed using the software “Primer3”, sequences are given in Table S2 (Supplementary Materials). Real-time PCR was performed with the SsoAdvanced Universal SYBR Green Supermix (BioRad, Vienna, Austria, #1725272) using the StepOnePlus instrument (Applied Biosystems, Foster City, CA, USA), and relative mRNA expression normalized to GAPDH. Fold changes in mRNA expression were calculated according to the 2-ΔΔCt method. Results are shown as mean fold induction of averaged Ct-values of triplicates.

### 2.6. Cell ELISA

**Figure 1:**
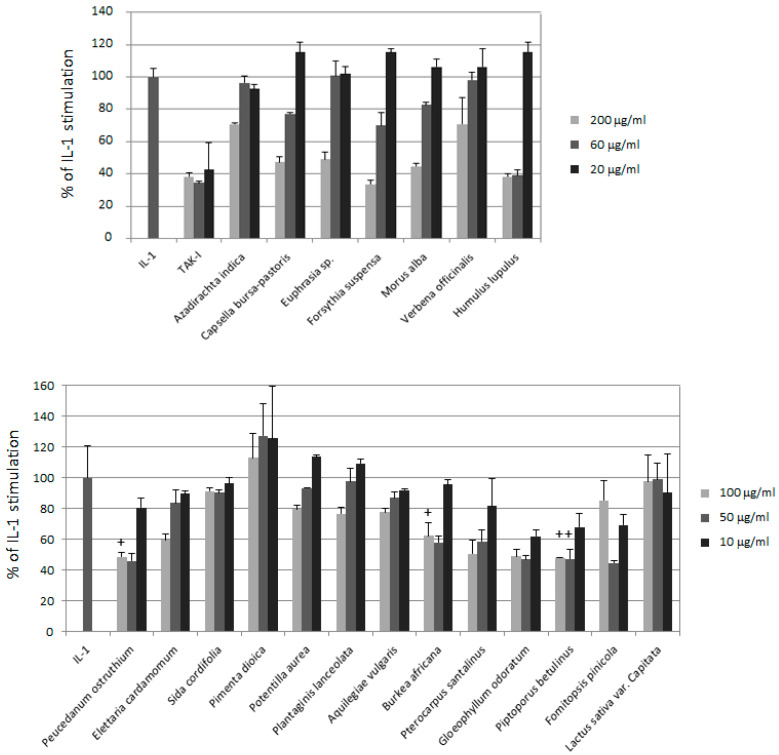
Inhibition of E-selectin expression by herbal and fungal extracts. Experiments were performed in two separate sets (upper and lower panel). HUVECs were pre-incubated for 30 min with different concentrations of extracts as indicated, stimulated with 5 ng/mL IL-1 for 4 h and analyzed by cell ELISA. The TAK inhibitor (TAK-I) was used as positive control at 10, 5, 2.5 μM. Relative levels of E-selectin as compared to IL-1 alone are shown. Samples marked with an “+” indicates apparent cytotoxicity as judged by morphological examination.

HUVECs were grown to post-confluency in 96-well plates, pre-treated for 30 min with various concentrations of the generated extracts as indicated in Figure 1, and then stimulated with IL-1ß (5 ng/mL). Experiments were performed in triplicate. After 4 h, cells were fixed and stained for E-selectin as described previously [20].

### 2.7. Western Blotting

Post-confluent HUVEC grown in 6-well plates were pre-incubated for 30 min with 50 μg/mL PO, stimulated with IL-1ß (5 ng/mL) for different periods of time (0, 10, 30, 90 min) and lysed in Laemmli buffer. Western blotting for IκBα, IKK2, their phosphorylated forms, as well as ß-actin, was done as described [21]. Densitometric analysis was done with Image J.

### 2.8. Transfection and Reporter Gene Assays

HUVEC were grown as described above and transfected with pNL3.2.NF-κB-RE (Promega) and pmaxGFP (Amaxa/Origene, Rockville, MD, USA) by electroporation using a BioRad Gene Pulser with the settings 200 V/960 μF. Then, 5 × 10^6^ cells were electroporated in 400 μL RPMI medium in 0.4 cm cuvettes with a total of 10 μg plasmid DNA. Cells were seeded into 6-well plates and grown for two days before stimulation. Luciferase levels were analyzed using the NanoGlo Luciferase Assay (Promega, #N1110) according to the manufacturer’s protocol and normalized to EGFP fluorescence.

### 2.9. Immunofluorescence Microscopy

Postconfluent HUVECs cultured on fibronectin-coated glass coverslips were treated with 50 ng/mL PO for 30 min prior to stimulation with 5 ng/mL IL-1ß. At the indicated timepoints, cells were fixed for 15 min with 4% paraformaldehyde (Sigma, #158127), permeabilized for 30 min with 0.1% Triton X-100 (Sigma, #93443), washed with PBS and finally blocked for one hour with 3% BSA-TBS-T. For immunostaining, rabbit polyclonal anti-p65 antibody (Santa Cruz, #sc-372) was used (1:500) with a secondary antibody, Alexa-Fluor 488-conjugated goat anti-rabbit IgG (Invitrogen, #A32723) at 1:1000. Cells were counterstained for 15 min with Alexa Fluor 568 phalloidin (Invitrogen, # A12380 1:1000) and 5 min with 4′,6-Diamidino-2-phenylindole (DAPI, Life Technologies, Carlsbad, CA, USA, #62247; 1:10000). Samples were examined with an Olympus IX71 microscope with a 20x/0.75 UPlanSApo objective. Images were processed with Image J software.

### 2.10. Ultra Performance Liquid Chromatography

UPLC analysis was performed on a Waters Acquity UPLC system (Waters Corporation, Milford, MA, USA; H-class) equipped with a quaternary solvent manager, a sample manager, a column manager, an isocratic solvent manager, a photodiode array (PDA) detector, an evaporative light scattering detector (ELSD), and a fraction collector. A Waters Acquity UPLC BEH Phenyl column (1.7 μm, 2.1 × 100 mm) was used for analytical experiments. Data acquisition and processing were conducted using the operating software Waters Empower 3. PO was chromatographed over UPLC using a binary mobile phase system consisting of (A) H2O and (B) CH_3_CN. The gradient was from 13–98% B in 12 min followed by 5 min re-equilibration. Method in detail: 13% B for 0.5 min, 13–18% B in 0.5 min, 18–45% B in 1 min, isocratic 45% B for 1.7 min, 45–73% B in 2.8 min, 73–98% B in 0.3 min, isocratic 98% B for 5 min, 98–13% B in 0.1 min, isocratic 13% B for 0.1 min; Conditions: temperature, 40 °C; flow rate, 0.300 mL/min; injection volume, 1 μL. Detection of compounds using PDA and ELSD. PDA conditions: 210 nm and full range spectra 192–400 nm. Further, the UPLC system was coupled to an Acquity QDa mass detector with an electrospray ionization source. An isocratic solvent manager was used as a make-up pump and positioned before the mass detector. The main flow stream was then split (1:10) and compounds detected in the positive ionization mode. (Ultrahigh-)gradient grade solvents from VWR Chemicals were used for all analytical experiments.

### 2.11. Statistical Significance Calculations

Differences between samples were analyzed by Ordinary one-way ANOVA with Dunnett´s multiple comparisons test using Graph Pad Prism software, San Diego, CA, USA. *, **, and *** indicate *p* < 0.05, 0.01, and 0.001, respectively.

## 3. Results

Within this study, natural materials from 17 plant species and three polypore species have been selected based on several criteria, primarily because of their long-standing traditional use in the field of inflammation and related areas, as well as anti-inflammatory effects described in the literature without in depth knowledge of their molecular mechanisms. Besides the 17 traditional herbal drugs, three polypore species were selected for the preparation of extracts, since they have been extensively used not only in TCM for the treatment of various ailments, but also for their health-promoting effects around the world [22]. A summary of the herbal and fungal materials used in this study, along with their traditional use and cognate pre-clinical and clinical studies, is given in Table 2.

Extracts of the selected herbal drugs and mushrooms were prepared to cope with a broad spectrum of metabolites endowed with drug like properties [11,12]. As a first assessment of their in vitro anti-inflammatory activity, we used a cell ELISA to screen these extracts for suppression of IL-1 stimulated E-selectin expression in human umbilical vein endothelial cells (HUVEC). The adhesion molecule E-selectin was chosen as a read-out, since it mediates one of the key steps in the inflammatory reaction, the initial weak adhesion (“rolling”) of leucocytes on the endothelial wall. As shown in Figure 1, many of the tested extracts inhibited E-selectin expression in a dose-dependent manner.

**Figure 2:**
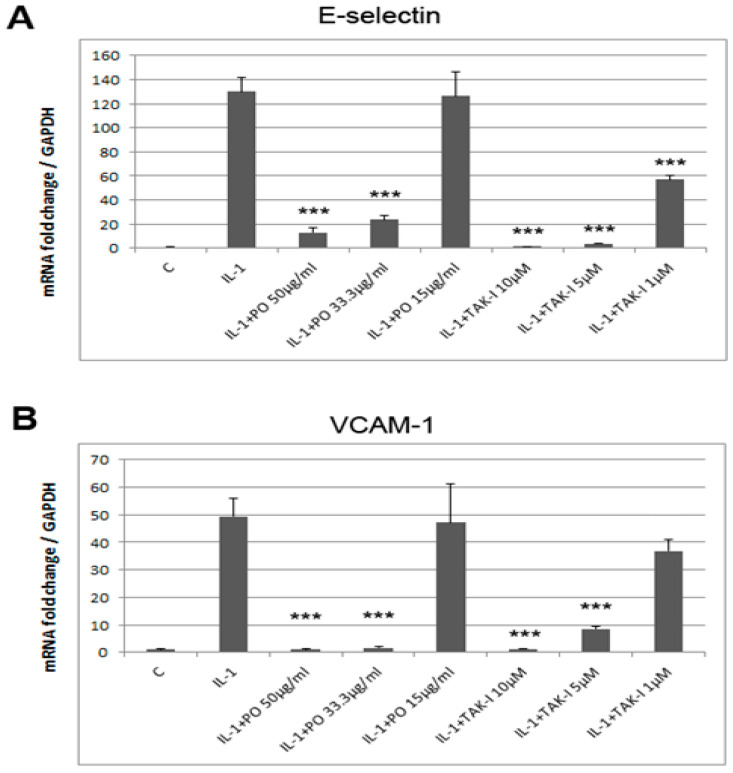
PO inhibits the expression of adhesion molecules in HUVEC. Post-confluent HUVEC were pre-incubated with different concentrations of PO for 30 min as indicated, then stimulated with IL-1 for 90 min and analyzed by qPCR for mRNA levels of (**A**) E-selectin and (**B**) VCAM-1. Values were normalized to GAPDH and are shown as fold change of IL-1 stimulation vs. unstimulated control. Triplicate samples were analyzed. *** *p* < 0.001 as compared to IL-1 stimulation. C: unstimulated control; TAK-I: positive control.

We selected the extract of *P. ostruthium* (PO) for further study based on the strength and robustness in repetitive experiments of its inhibitory effect. PO was characterized by PDA/ELSD-UPLC, and by means of UPLC-ESI-MS its main constituents could be dereplicated (Figure S1; Supplementary Materials). In Table S1 (Supplementary Materials) the results from the dereplication via literature search in SciFinder (accessed 2020/05/11) are presented. As a first step into the mechanistic investigation of E-selectin expression, we analyzed the mRNA levels after pre-incubation of HUVEC with different concentrations of PO (i.e., 50, 25 and 12 μg/mL, respectively) and stimulation with 5 ng/mL IL-1. In addition, another adhesion molecule, VCAM-1, which mediates the firm adhesion of leucocytes to the endothelium, was analyzed. The choice of pre-incubation time was done based on previous experience with herbal extracts [20] and on experiments analyzing different times of pre-incubation depending on the concentration (Figure S2; Supplementary Materials). PO did not show toxicity at these concentrations (Figure S3; Supplementary Materials). As shown in Figure 2, PO strongly inhibited the expression of both adhesion molecules on the mRNA level in a dose-dependent manner, ranging from 50 to 15 µg/mL.

**Figure 3:**
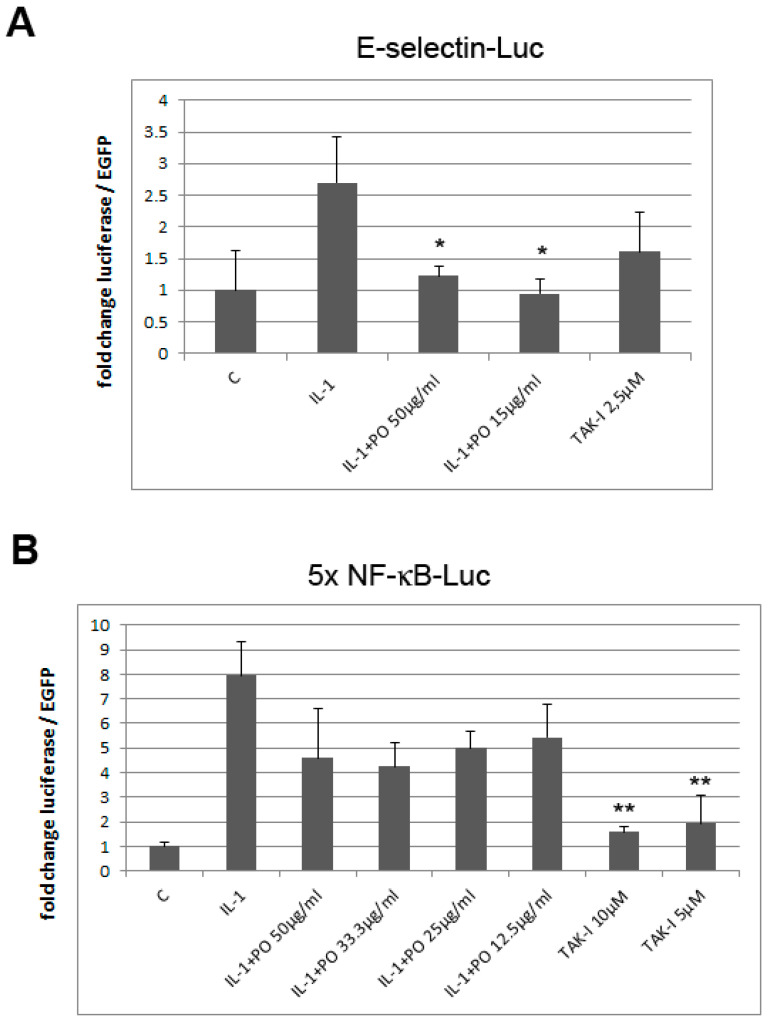
PO inhibits E-selectin promoter and NF-κB activity. HUVEC were transfected with either an E-selectin (**A**) or a NF-κB luciferase reporter construct (5xNF-κB luc; (**B**)) and EGFP as internal control, pretreated with PO (or TAK inhibitor as positive control) and stimulated with IL-1 as indicated. Luciferase levels were determined after 16 h and are shown as relative levels normalized to EGFP. Triplicate samples were analyzed. * *p* < 0.05, ** *p* < 0.01 as compared to IL-1 stimulation; C: unstimulated control.

Since mRNA levels can be regulated on the level of transcription but also depend on other factors such as mRNA stability, we then determined whether the inhibition of E-selectin expression occurs on the transcriptional level. HUVEC were transfected with an E-selectin promoter luciferase reporter construct and, two days later, after reaching post-confluency, they were pre-incubated with PO and stimulated with IL-1. As shown in Figure 3A, PO inhibited the activity of the E-selectin promoter. Since the expression of E-selectin (and also VCAM-1) is dependent on NF-κB, we investigated whether PO might act via inhibition of this transcription factor. Therefore, HUVEC were transfected with a construct containing a multimerized NF-κB binding site driving the expression of a luciferase reporter gene, treated as above and analyzed for luciferase expression. PO diminished NF-κB activity at all concentrations tested (although statistical significance was not reached; Figure 3B).

**Figure 4:**
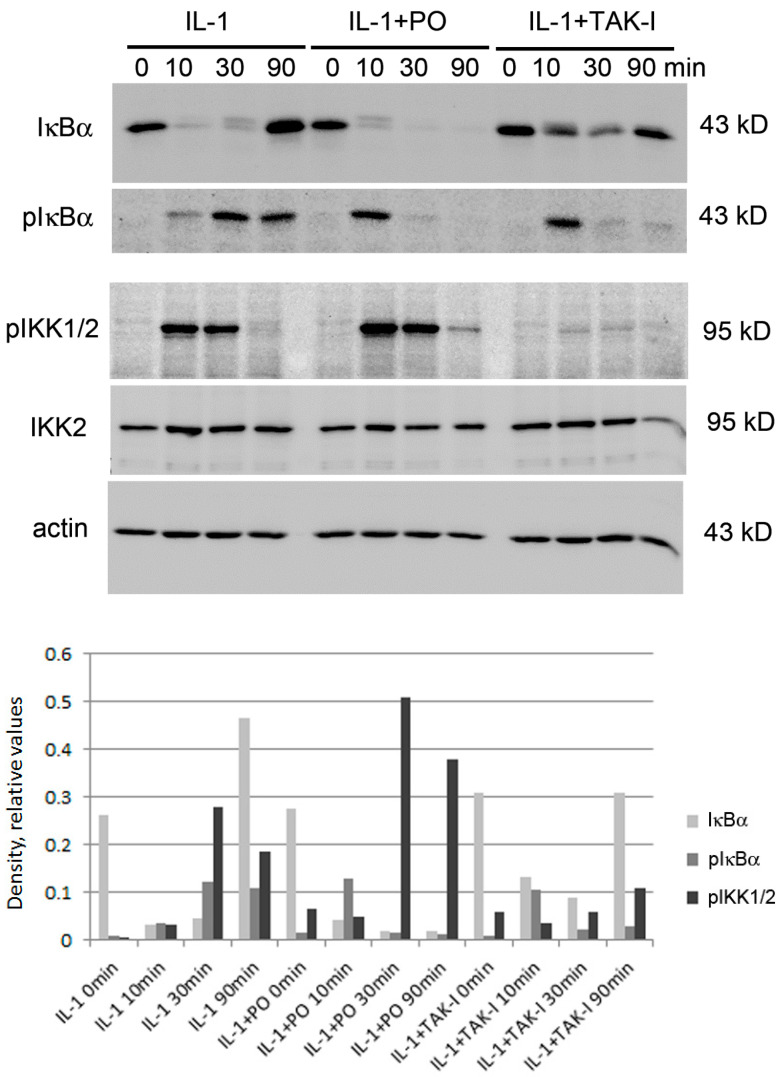
PO affects IκBα degradation and IKK phosphorylation. Post-confluent HUVEC were pre-incubated with 50 μg/mL of PO for 30 min, then stimulated with 5 ng/mL IL-1 for 90 min and probed for IκBα, phospho-IκBα, and phospho-IKK1/2; IKK2 and ß-actin served as loading controls, the TAK-inhibitor (5 μM) as control for inhibition. IκBα/pIκBα and IKK2/pIKKs/actin were analyzed on separate gels. A densitometric quantification is shown below.

Since one of the key steps in NF-κB signaling is the phosphorylation and degradation of its inhibitor IκBα, which takes place within minutes after stimulation and is followed by re-synthesis that starts approx. one hour later [20,119], we assayed IκBα and phospho-IκBα levels by Western blotting. HUVEC were pre-incubated and stimulated as above, except that the times of stimulation were adjusted to assay for both the early and late events. As shown in Figure 4, IκBα was degraded within 10 min following IL-1 stimulation and re-synthesized at 90 min; in PO-treated cells, degradation was slightly delayed, but re-synthesis entirely inhibited. Accordingly, and in contrast to IL-1 stimulation alone, IκBα phosphorylation was still visible at 10 min in IL-1+PO-treated cells, suggesting a delay in degradation. Previously, the group of D. Baltimore described “oscillations” of NF-κB nuclear-cytoplasmic translocation due to several rounds of degradation and re-synthesis of IκBα as well as other IκB species [120]. Whereas this phenomenon can be seen best in T cells where multiple waves can be observed, endothelial cells also show at least one additional wave of NF-κB nuclear translocation and the associated IκBα phosphorylation and degradation. In our experiments, this second wave is indicated by the re-appearance of pIκBα between 30 and 90 min; it is lacking in PO (as well as in TAK-I) pretreated cells. In addition, the phosphorylation of IKKs in the activation loop, which is a measurement for their activity [121], did not show, besides a weak prolongation, a pronounced difference between IL-1- and IL-1+PO-treated HUVEC; together, these data indicate that the initial steps of activation, namely IKK activation and IκBα phosphorylation/degradation, are weakly affected, whereas the later steps are predominant. This suggests that in HUVEC, PO displays two types of activity towards NF-κB, one (weaker) that affects the initial steps of activation, and a second, more pronounced one that is directed towards a downstream part of the NF-κB signaling cascade.

**Figure 5:**
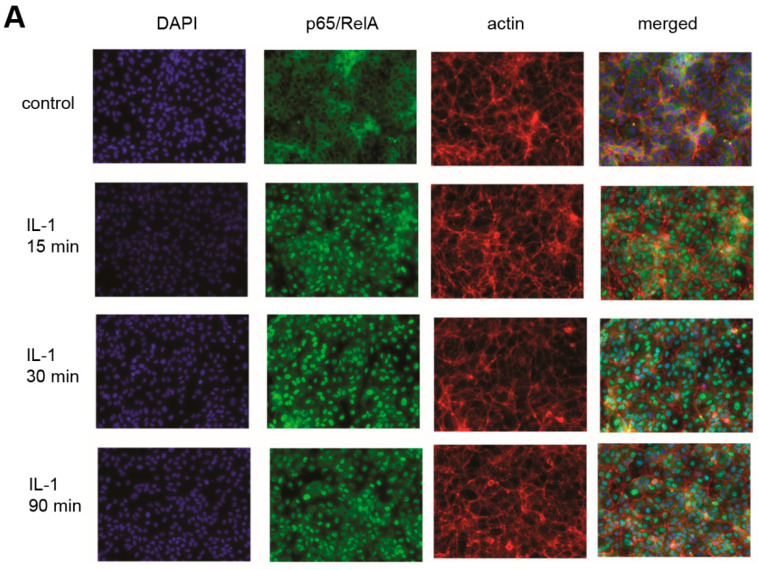

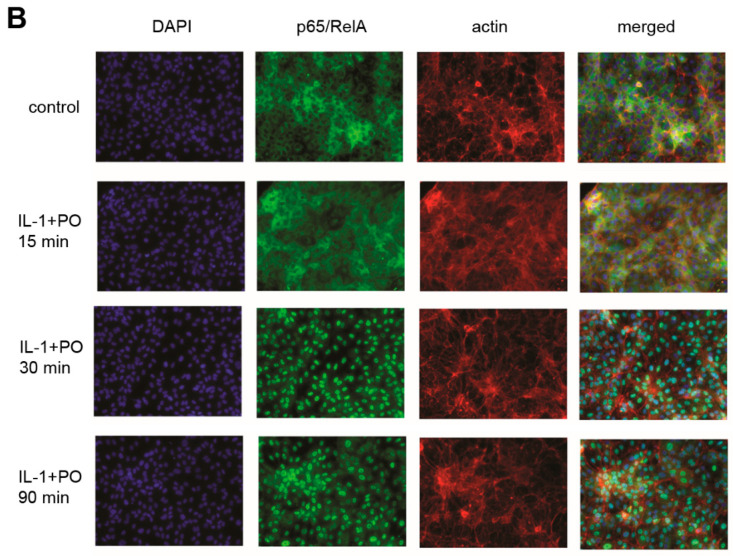
PO delays nuclear translocation of NF-κB. HUVEC were either (**A**) stimulated with 5 ng/mL IL-1 alone for the indicated times or (**B**) pre-treated for 30 min with 50 ng/mL PO before IL-1 stimulation, and immunostained for the p65/RelA subunit of NF-κB (green) and actin (red). Nuclei were stained with DAPI (blue). Merged pictures are shown on the right.

In order to further analyze the mechanism and kinetics of PO on NF-κB activation, we performed immunostaining to asses NF-κB nuclear translocation. HUVEC were pre-treated as above, and stimulated with 5 ng/mL IL-1 for 15, 30, and 90 min. As shown in Figure 5A, the p65 subunit of NF-κB (RelA) translocated into the nucleus within 15 min, whereas this was delayed in PO-treated cells (Figure 5B). The later time points were not affected. This confirmed that PO does have an effect on an early step of NF-κB signaling, but that additional mechanisms at later stages of the signaling cascade are operative, that add to the later events such as the lack of IκBα re-synthesis, the overall diminished activity in the reporter gene assay and, as a result, diminished adhesion molecule expression.

## 4. Discussion

Based on the long-standing traditional use of masterwort in the Alpine region and further studies in the scientific literature, but limited knowledge of its mode of action, we selected PO from a set of extracts with anti-inflammatory activity for further analysis in our model system. *Peucedanum* (Apiaceae) comprises approximately 120 species in several continents including Europe, Africa, Asia, and North America. Many of these have been used in traditional medicine for the treatment of a variety of diverse disorders, e.g., pyrexia, cardiovascular, gastrointestinal or neurological diseases. This is likely due to the broad spectrum of constituents, including various coumarins, phenolic acids, amines, glycosides, flavonoids, diterpenes and components of their essential oils [122].

Regarding inflammatory conditions, mainly *P. praeruptorium, P. japonicum*, and *P. decursivum* have been subject to more intense research, often in the context of airway inflammation [123,124,125,126]. PO contains a variety of furanocoumarins including imperatorin and iso-imperatorin, pimpinellin and iso-pimpinellin, oxypeucedanin and derivatives, ostruthol, peucedocoumarin III, coumarines such as ostruthin and the chromones peucenin and alsaticol [83,122]. PO extracts and isolated compounds have been found to inhibit the PIK3/Akt/mTor pathway [127], prevent SMC proliferation [86], and display anti-Amyloidß1-42 aggregation [84], anti-mycobacterial and anti-insecticidal activities [128]. Anti-inflammatory and immunomodulatory activities were attributed to the coumarin family member osthole and its derivatives [129]. Moreover, Hiermann and Schantl reported antiphlogistic and anti-pyretic effects as well as the inhibition of cyclooxygenase and 5-lipoxygenase by PO extracts and isolated coumarin compounds [85].

Only recently, we identified and isolated several bioactive compounds from PO by applying a novel biochemometric approach named Eliciting Nature’s Activities (ELINA; [18,130]) with an NF-ĸB reporter-gene assay plus two assays for E-selectin and VCAM-1 as biological read-out. However, the anti-inflammatory activity could only partially recovered (as compared to the total extract) by the combination of imperatorin and peucenin as a mixture, suggesting additional combinatorial effects [18].

Therefore, we here investigated the anti-inflammatory activity of the total extract in more detail and provide first insight into its molecular mechanism(s) of action. In IL-1 stimulated endothelial cells, PO suppressed the expression of the adhesion molecule E-selectin. Inhibition was found on both the mRNA and protein levels. Moreover, PO inhibited the activity of E-selectin on the transcriptional level, as demonstrated by reporter gene experiments (Figure 3A). Moreover, since E-selectin transcription is dependent on NF-κB, we also investigated the activity of PO on this transcription factor by reporter gene analysis and demonstrate that PO strongly inhibited NF-κB activity (Figure 3B).

One of the key steps in the NF-κB signaling pathway is the phosphorylation and ubiquitin-dependent degradation of the inhibitor IκBα, which forms a cytoplasmic complex with NF-κB p65/p50 to prevent its nuclear translocation. In contrast to IL-1 alone, IL-1 in combination with PO treated cells showed a slight delay in IκBα phosphorylation and degradation, and a total lack of IκBα re-synthesis (which is NF-κB-dependent). The re-appearance of phospho-IκBα specific species at 30 and 90 min after treatment, indicative of a second wave of NF-κB activation, is lacking in PO-treated cells. This second wave has been observed in other cells, and was most pronounced in T cells where even multiple oscillations can occur that result from repeated degradation and resynthesis in the NF-κB/IκBα (and other IκBs such as -ß and -e) system [120]. It has been speculated that these have differential effects on the expression of NF-κB-dependent genes [131].

To investigate upstream events, we additionally probed for activation of IKK2, the main kinase responsible for IκBα phosphorylation and found that, in line with the IκBα data, its activation was only slightly inhibited. In contrast, our positive control (5Z)-7-oxozeaenol that inhibits TAK1, a kinase that together with its co-activators TAB1 and -2/3 transmits the signal from the IL-1 (and also TNF) receptor to activate IKK2, completely prevented IKK2 phosphorylation and subsequent IκBα degradation. Together, it can be concluded that PO displays a weak effect on either IKK2 or an upstream signaling molecule, but that additional more downstream effects have to be operative.

This was subsequently confirmed by immunofluorescence studies, which showed a delay in nuclear translocation of NF-κB (Figure 5, compare 15 min timepoint between IL-1 (A) and IL-1+PO, (B)). Surprisingly, however, at 30 and 90 min, NF-κB nuclear translocation was indistinguishable between these conditions. This suggests that, at these timepoints, although NF-κB resides in the nucleus, its activity must be impaired by other mechanisms. At this point, we can only speculate about their nature, but several possibilities have been described, e.g., post-translational mechanisms such as phosphorylation, acetylation, S-nitrosylation that affect either DNA-binding or transactivation, miRNAs, or the crosstalk with other signaling pathways such as PPARα and -ß, ATF3, or STAT3. These will be the subject of further investigations.

## 5. Conclusions

The anti-inflammatory activity that has been ascribed to PO by traditional medicine and previous reports could be substantiated in this study and attributed to the inhibition of gene expression of the pro-inflammatory cell adhesion molecules E-selectin and VCAM-1. Thereby, PO acts by inhibiting predominantly, but probably not exclusively, the activity of NF-κB. Although this inhibition does involve, to a certain degree, the initial steps of activation including nuclear translocation, a major part of the inhibitory activity takes place at later stages. From a therapeutic point of view NF-κB inhibition holds promise for the treatment of a wide variety of mainly inflammation-related disorders, but also others such as, e.g., therapy-resistance in cancer. However, most cognate NF-κB inhibitors target signaling molecules that act early in the pathway such as IKK2, TAK1, or IκBα (through inhibition of ubiquitination). Our observation that PO also inhibits later stages of activation could be of interest, since it suggests that it affects signaling molecules which are distinct from cognate drug targets. Identification of these may open the way for the identification of lead compounds and, subsequently, novel drugs.

## Figures and Tables

**Table 1:** Information on used natural materials and extracts (LLE, lead-like enhanced extracts; LLE w/o TD, lead-like enhanced extracts without tannin depletion).

Species	Family	Organ	Provider/Collection Site	Voucher Specimen/Charge Number	Used Extract
*Aquilegia vulgaris* L.	Ranunculaceae	herb	Padma AG, Wetzikon, Switzerland	Ch.Nr.: 21290300	LLE
*Azadirachta indica* A.Juss.	Meliaceae	fruits	Padma AG, Wetzikon, Switzerland	JR-20150615-A1 Ch.Nr.: 2021108301	LLE
*Burkea africana* Hook.	Leguminosae	bark/heart-wood	Northwest of Zeerust, South Africa	Voucher no 5858 [14]	Ethanol (EtOH) extract
*Capsella bursa-pastoris* (L.) Medik.	Brassicaceae	herb	Kottas Pharma GmbH, Vienna, Austria	Ch.Nr.: 1223296	MeOH
*Elettaria cardamomum* (L.) Maton	Zingiberaceae	fruits	Padma AG, Wetzikon, Switzerland	Ch.Nr.: W17201998	LLE w/o TD
*Euphrasia officinalis* L.	Orobanchaceae	herb	Kottas Pharma GmbH, Vienna, Austria	JR-20090625-A1 Ch.Nr.: KLA90309	LLE
*Fomitopsis pinicola* (Sw.) P. Karst. (strain 10)	Fomitopsidaceae	fruit body	Viggartal, Ellbögen, Austria (grown on dead spruce trunk); Ursula Peintner	FompinE0010	LLE w/o TD
*Forsythia suspensa* (Thunb.) Vahl	Oleaceae	fruits	Plantasia GmbH, Oberndorf bei Salzburg, Austria	Ch.Nr.: 020410	LLE
*Gloeophyllum odoratum* (Wulfen) Imazeki (strain 54)	Gloeophyllaceae	fruit body	Oberperfuss, Austria (grown on spruce) Ursula Peintner	JR-20140310-A1 GloodoE0054	EtOH extract [17]
*Humulus lupulus* L.	Cannabaceae	flower	Kottas Pharma GmbH, Vienna, Austria	Ch.Nr.: W12203440	CH_2_Cl_2_
*Lactuca sativa* L.	Compositae	leaves	Padma AG, Wetzikon, Switzerland	Ch.Nr.: 21400300	LLE
*Morus alba* L.	Moraceae	root bark	Plantasia GmbH, Oberndorf bei Salzburg, Austria	Ch.Nr.: 710797	LLE
*Peucedanum ostruthium* (L.) W.D.J.Koch	Apiaceae	roots/rhizom	Birgitzköpfl, Axamer Lizum, Austria	JR-20120814-A1 (small scale extract)	LLE
*Peucedanum ostruthium* (L.) W.D.J.Koch	Apiaceae	roots/rhizom	Kottas Pharma GmbH, Vienna, Austria	JR-20180119-A2 Ch.Nr.: P17301770 (large scale extract)	LLE w/o TD [18]
*Pimenta dioica* (L.) Merr.	Myrtaceae	fruits	Padma AG, Wetzikon, Switzerland	JR-20150615-A4 Ch.Nr.: 21362100	LLE
*Piptoporus betulinus* (Bull.) P. Karst. (strain 39)	Fomitopsida-ceae	fruit body	Vahrn bei Brixen, Italy, grown on birch, Ursula Peintner	PipbetE0039	LLE w/o TD
*Plantago lanceolate* L.	Plantaginaceae	herb	Padma AG, Wetzikon, Switzerland	Ch.Nr.: 21327101	LLE
*Potentilla aurea* L.	Rosaceae	herb	Padma AG, Wetzikon, Switzerland	JR-20150615-A3 Ch.Nr.: 21161301	LLE
*Pterocarpus santalinus* L.f.	Leguminosae	wood	Padma AG, Wetzikorn, Switzerland	JR-20190315–A1 Ch.Nr.: P16301836	LLE
*Sida cordifolia* L.	Malvaceae	herb	Padma AG, Wetzikon, Switzerland	JR-20150615-A2 Ch.Nr.: 20981300	LLE
*Verbena officinalis* L.	Verbenaceae	herb	Ehnbach-klamm, Zirl, Austria	JR-20120801-A12	LLE

**Table 2:** Herbal and fungal materials with anti-inflammatory activity selected for this study, including their traditional application and reported in vitro and in vivo data; clinical evidence also includes studies not directly linked to inflammation.

Herbal/Fungal Material	Traditional Use	In Vitro/In Vivo Studies	Clinical Studies
Herbs of *Aquilegia vulgaris* (European columbine)	Southern Europe, Asia and Africa; chronic skin inflammation, liver and bile duct disorders, jaundice [23,24,25,26].	Hepatoprotective [24,26] and antioxidant effects [23,25].	
Fruits of *Azadirachta indica* (Neem fruits)	Medicinal systems (Ayurveda [27,28], Unani and Siddha [29]); as astringents and antihelmintic agent [30].	Antioxidant and neuroprotective effecs [27]; anti-bacterial [31], anti-nociceptive [32] and osteogenic activities [33].	
Roots and bark of *Burkea africana* (Seringa tree)	Sub-Saharan Africa; Pain (e.g., headache and migraine), inflammation and as wound-healing agents [34].	Antioxidant [34] and anti-viral activites [14,15].	
Herbs of *Capsella bursa-pastoris* (Shepherd’s purse)	Traditional Chinese [35] and Korean folk medicine; treatment of hypertension and edema [36].	Anti-inflammatory [35,36,37] and anti-viral activities [15].	Reduced postpartum hemorrhage bleeding in women [38].
Fruits of *Elettaria cardamomum* (Queen of spices)	Ayurveda, Siddha and Unani [39]; Treatment of asthma, teeth infections, cataracts, digestive and kidney disorders [40].	Anti-inflammatory [41,42,43], anti-tumor [42,44], anti-microbial [41,45] activities;Immunomodulatory effects [42].	Improved parameters of inflammation and oxidative stress in pre-diabetic women [46].
Herbs of *Euphrasia officinalis* (Eyebright herb)	Anthroposophical medicine [47]; conjunctivitis, ophthalmia and ocular allergies [48]	Anti-inflammatory activity [37,49]; reduction of UVB-induced cell death and increased wound-healing ability [50].	Effective against allergic conjunctivitis and conjunctivitis due to external irritants or other causes [47].
Fruit bodies of *Fomitopsis pinicola* (Red banded polypore)	Traditional Asian [51] and European medicine; Nausea, headache and liver problems [22].	Anti-tumor [52,53], anti-oxidant [54], anti-bacterial [55] and anti-inflammatory activities [56,57].	
Fruits of *Forsythia suspensa* (Lian Qiao)	Traditional Chinese Medicine (TCM) as heart-clearing and detoxifying agent [58,59,60].	Anti-inflammatory [59,61,62], anti-tumour [59,63], antioxidant [64] and anti-viral activities [15,58,60].	
Fruit bodies of *Gloeophyllum odoratum* (Anise mazegill)	Central Europe, Asia and North America [17,65]	Anti-influenza virus activity [17]; antioxidant [66] and anti-cancer activities [67]; Thrombin inhibition [68].	
Female inflorescences of *Humulus lupulus* (Hops)	Management of sleeping disorders, as sedative, and bitter stomachic [69,70].	Anti-inflammatory [71,72], antioxidant activities; hepatoprotective [73] and osteoprotective effects [74].	Effective against mild depression, anxiety and stress [75]; reduction of early postmenopausal symptoms (hot flashes, sexual dysfunction, anxiety and depression) [76].
Leaves of *Lactuca sativa* (Butterhead lettuce)	Popular vegetable; Perception as “healthy” food [77].	Antioxidant properties [78,79].	
Root bark of *Morus alba* (Sang-Bai-Pi)	TCM; Throat infections, asthma, fever and inflammation [80].	Antioxidant, anti-cancer, anti-inflammatory [80,81,82] and anti-viral activities [15].	
Roots and rhizome of *Peucedanum ostruthium* (Masterwort)	Austrian and Italian folk medicine; gastro-intestinal, cardiovascular and respiratory diseases [37,83,84,85].	Anti-inflammatory activity [37,85]; inhibition of vascular smooth muscle cell proliferation [86]; anti-amyloidogenic activity [84].	
Fruits of *Pimenta dioica* (Allspice)	Jamaican [87] and Costa Rican medicine; menopausal symptoms, dysmenorrhea and dyspepsia [88].	Anti-oxidant [87], anti-diabetic effects [89] and metabolic disorders via upregulation of TGR_5_ [90]; oestrogenic effects, inhibitory effect on breast and gastric cancer cells [88].	
Fruit bodies of *Piptoporus betulinus* (Birch polypore)	Central European folk medicine [22]; fatigue and immune-enhancing properties [91]	Anti-microbial [92], anti-inflammatory [91] and anti-cancer activities [93].	
Leaves of *Plantago lanceolata* (Ribwort plantain)	Slovakian and Southeast European folk medicine; gastric ulcers, respiratory infections and wound healing [94,95].	Anti-inflammatory activities [36]; improved skin wound healing [95].	
Herbs of *Potentilla aurea* (Goldfinger herb)	Southern and Central Europe [96,97,98], homeopathic medications; diarrhea, diabetes mellitus and inflammations [99];	Pancreatic lipase and α-amylase inhibition [100]; anti-urease activity [101]	
Heartwood of *Pterocarpus santalinus* (Red sanders)	India and Korea; Inflammation, mental aberrations, cancer and ulcer [102,103]	Antioxidant properties; anti-microbial [104] and anti-inflammatory activities [103,105].	
Herbs of *Sida cordifolia* (Indian mallow)	Indian, Chinese, African and Brazilian medicine; skin diseases [106,107], inflammation of oral mucosa, nasal congestion, asthmatic bronchitis and rheumatism [108].	Anti-nociceptive [106], anti-inflammatory [109], antioxidant activity and improved wound healing [107]; Hepatoprotective effects [108].	
Herbs of *Verbena officinalis* (Vervain)	European [110] and Chinese folk medicine; rheumatism and bronchitis [111].	Anti-oxidant [112,113,114], anxiolytic, sedative, anti-convulsant effects [115,116]; anti-inflammatory activities [37,117].	Ameliorative effect of chronic generalized gingivitis [118].

## Supplementary Materials

The following are available online at https://www.mdpi.com/2218-273X/10/9/1215/s1, Figure S1: UPLC of PO showing the (A) total ion chromatogram (TIC, positive mode) containing compounds 1–8, (B) ELSD and (C) PDA 210 nm chromatogram; Figure S2: Pre-incubation time with PO affects its inhibitory potential; Figure S3: Cytotoxicity of PO; Table S1: Results from the dereplication of PO via literature search; Table S2: Primers for real-time PCR.
