# Supplementary material for: Peucedanum ostruthium Inhibits E-Selectin and VCAM-1 Expression in Endothelial Cells through Interference with NF-κB Signaling
Source: Biomolecules. 2020 Aug 21;10(9):1215. doi: 10.3390/biom10091215 (PMC7563923; doi:10.3390/biom10091215)
Supplement: Supplementary file 1 [file biomolecules-10-01215-s001.pdf]

biomolecules

Article

Peucedanum ostruthium inhibits E-selectin and VCAM-1 expression in endothelial cells through interference with NF-κB signaling.

Christoph Lammel [1], Julia Zwirchmayr [2], Jaqueline Seigner [1], Judith M. Rollinger [2,*], and Rainer de Martin [1]

[1] Department of Vascular Biology and Thrombosis Research, Medical University of Vienna, Schwarzspanierstaße. 17, 1090 Vienna, Austria
[2] Department of Pharmacognosy, Faculty of Life Sciences, University of Vienna, Althanstraße 14, 1090 Vienna, Austria
* Correspondence: judith.rollinger@univie.ac.at; Tel.: +43-1-4277-55255; Fax: +43-1-4277-855255

Received: date; Accepted: date; Published: date

Figure S1

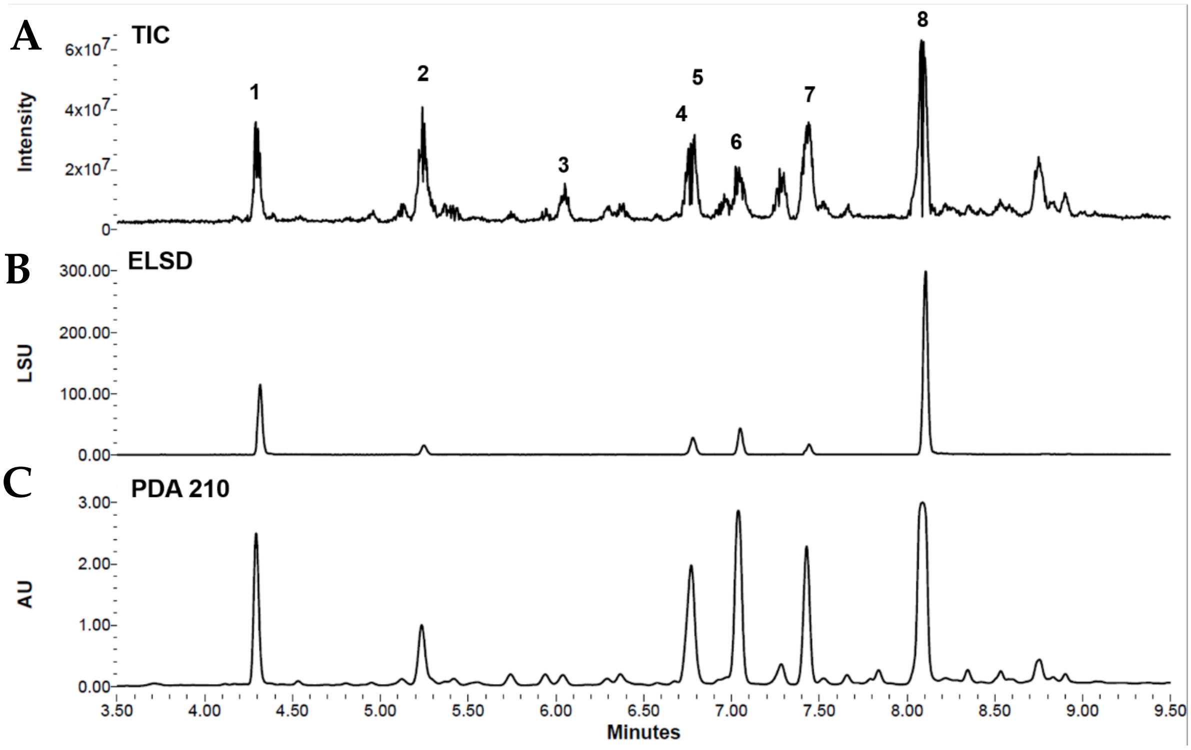

Figure S1 UPLC of PO showing the (**A**) total ion chromatogram (TIC, positive mode) with annotated compounds **1** – **8** (Table S1), (**B**) ELSD chromatogram, and (**C**) PDA 210 nm chromatogram.

Figure S2

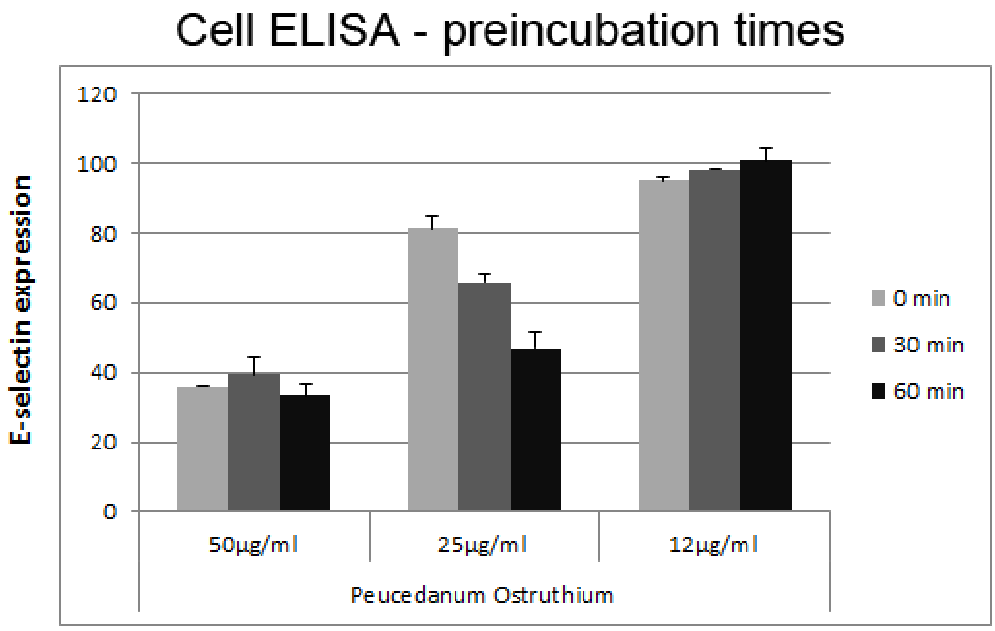

Figure S2 Pre-incubation time with PO affects its inhibitory potential.

Figure S3

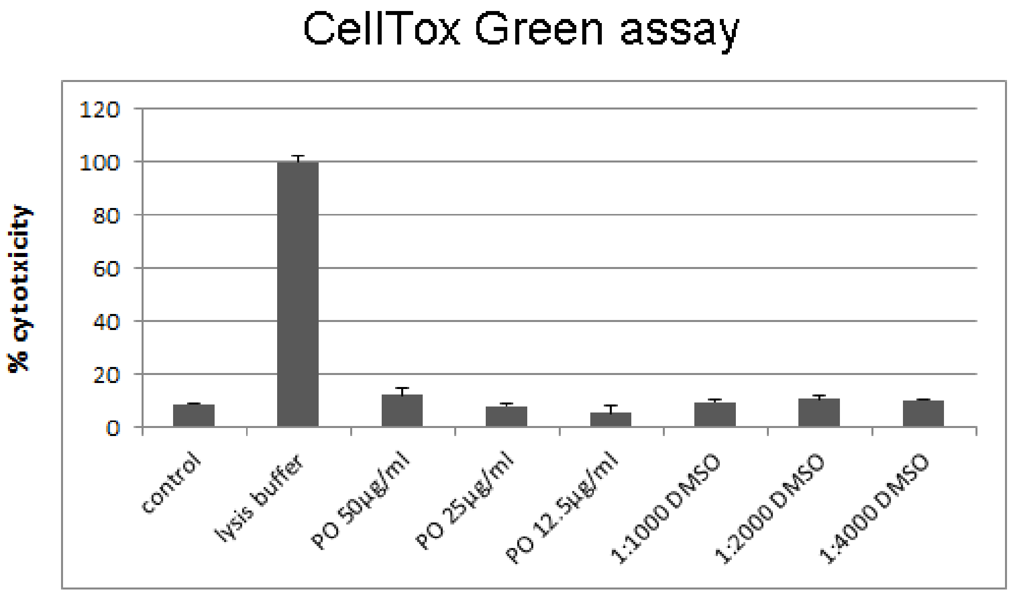

Figure S3 Cytotoxicity of PO extract.

Table S1 Results from the dereplication of PO via literature search

	Retention time [min]	*m/z* value [positive mode]	Estimated molecular weigth [g/mol]	Proposed compound	CAS Registry Number
**1**	4.293	305.05	304.0	oxypeucedanin hydrate	2643-85-8
**2**	5.245	319.04	318.0	oxypeucedanin methanolate	52939-12-5
**3**	6.039	287.09	286.0	oxypeucedanin	26091-73-6
**4**	6.747	261.15	260.1	peucenin	578-72-3
**5**	6.784	387.11	386.1	ostruthol	642-08-0
**6**	7.040	271.13	270.1	imperatorin	482-44-0
**7**	7.420	271.11	270.1	isoimperatorin	482-45-1
**8**	8.066	299.13	298.1	ostruthin	148-83-4

Table S2 Primers for real-time PCR

Name	Forward (5´-3´)	Reverse (5´-3´)
glyceraldehyde 3-phosphate dehydrogenase (GAPDH)	AGAAGGCTGGGGCTCATTT	CTAAGCAGTTGGTGGTGCAG
E-selectin (SELE)	CCTGTGAAGCTCCCACTGA	GGCTTTTGGTAGCTTCCATCT
vascular cell adhesion molecule 1 (VCAM1)	CCGGCTGGAGATATTAC	TGTATCTCTGGGGGCAACAT

© 2020 by the authors. Submitted for possible open access publication under the terms and conditions of the Creative Commons Attribution (CC BY) license (http://creativecommons.org/licenses/by/4.0/).
